# Mutation in *Phex* Gene Predisposes BALB/c-*Phex^Hyp-Duk^/Y* Mice to Otitis Media

**DOI:** 10.1371/journal.pone.0043010

**Published:** 2012-09-28

**Authors:** Fengchan Han, Heping Yu, Ping Li, Jiangping Zhang, Cong Tian, Hongbo Li, Qing Yin Zheng

**Affiliations:** 1 The Transformative Otology and Neuroscience Center, Binzhou Medical University, Yantai, Shandong, People's Republic of China; 2 Department of Otolaryngology-Head and Neck Surgery, Case Western Reserve University, Cleveland, Ohio, United States of America; 3 The Jackson Laboratory, Bar Harbor, Maine, United States of America; Whitehead Institute, United States of America

## Abstract

Genetic susceptibility underlying otitis media (OM) remains to be understood. We show in this study that mutation in *Phex* gene predisposes the BALB/c-*Phex^Hyp-Duk^/Y* (abbreviated *Hyp-Duk/Y*) mice to OM, which occurs at post-natal day 21 (P21) with an average penetrance of 73%. The OM was identified by effusion in the middle ear cavity and/or thickening of middle ear mucosae, and was characterised by increase in goblet cells, deformity of epithelial cilia and higher expression of proliferating cell nuclear antigen (PCNA) in cells of the middle ear mucosae. Moreover, the transcription levels of *Tlr2, Tlr4, Nfkb1, Ccl4, Il1b* and *Tnfα* in the ears of the *Hyp-Duk/Y* mice at P35 were significantly upregulated, compared to those of the controls. Higher expression levels of TLR2, TLR4, NF-κB and TNF-α in the middle ears were demonstrated by immunohistochemistry (IHC). However, the OM in the mice was not prevented by azithromycin administration from gestational day 18 to P35. Further study showed that, in contrast to the low mRNA levels of *Phex* gene in the ears of the *Hyp-Duk/Y* mice, the mRNA level of *Fgf23* was significantly elevated at P9, P14, P21 and P35. Meanwhile, mRNA levels of EP2 (PGE2 receptor), which expressed in the middle ear epithelia as demonstrated by IHC, were already increased at P14 even before the occurrence of OM, indicating that PGE2, an inflammatory mediator, is involved in the OM development in the mutants. Taking together, *Phex* mutation primarily up-regulates gene expression levels in FGF23 mediated pathways in the middle ears, resulting in cell proliferation and defence impairment at the mucosae and subsequently bacterial infection. The *Hyp-Duk/Y* mouse is a new genetic mouse model of OM.

## Introduction

Otitis media (OM) continues to be one of the most common childhood infections [1]. Human predisposition to OM is affected by multiple factors, including Eustachian tube (ET) structure and function, immune status, innate mucosal defence, genetic susceptibility and pathogen exposure [1]. Increasing evidence supports an important role for heredity in susceptibility to OM [2]. Animal models with OM linked to a defined genetic lesion are important to reveal pathogenesis and underlying genetic pathways linked to OM [3].

X-linked hypophosphatemic rickets (XLH) is a dominant disorder characterised by hypophosphatemia resulting from impaired renal tubular reabsorption of inorganic phosphate [4]. In humans, XLH is caused by mutation in the *Phex* gene which codes for a 749-amino acid protein. Five mutations in the mouse *Phex* gene have been reported: *Hyp, Hyp-2J, Hyp-Duk, Gy*, and *Ska1*. The *Hyp-Duk* mutation is a spontaneous intragenic deletion involves at least 30 kb containing *Phex* exons13 and 14 as indentified by Southern blot analysis [5]. Because of the common phenotypes (including small body size, slightly abnormal skull shape and short tail) among *Phex^Hyp-Duk^/Y* (*Hyp-Duk/Y*) mutants, it can be inferred that the mutation results in loss of PHEX protein function in the ears. Actually, auditory brainstem response (ABR) threshold analysis showed that *Hyp-Duk/Y* mice have substantial hearing impairment compared to normal controls and to other *Phex* mutants [5].

In this investigation, we further characterise *Hyp-Duk/Y* hemizygotes, which present a high incidence of chronic OM. We present evidence of OM in the mutants and a possible mechanism for OM development. Our data imply that *Phex* gene mutation predisposes *Hyp-Duk/Y* mice to OM primarily by FGF23 mediated pathways. OM in the *Hyp-Duk/Y* mice may ultimately cause conductive, and possibly sensory, hearing loss.

## Materials and Methods

### Mice and animal care

The BALB/cAnBomUrd-*Phex^Hyp-Duk^* mice were originally housed in The Jackson Laboratory (Bar Harbor, ME, USA) research facilities and were relocated to Case Western Reserve University and maintained through sibling inbreeding. Mainly male mice, mutants (*Hyp-Duk/Y*, n = 122) and controls (*+/Y*, n = 109) ranging in age from post-natal day 9 (P9) to 20 weeks, were included in this study. All mice were genotyped by PCR using conditions and primers described previously [6]. Care and use of animals were approved by the Institutional Animal Care and Use Committee of The Jackson Laboratory (TJL, Bar Harbor, ME) and of Case Western Reserve University.

**Table 1:** Sequence of RT-PCR primers.

ID	Sequence	Product Size (bp)	Reference
PhexF1	5′-AAGCTGGACCAAGCAACACT-3′ (in exon 5)		
PhexR1	5′-TCGGTTCTCATGTGGAATCA-3′ (in exon 8)	213	
Phex2F	5′-AGGCATCACATTCACCAACA-3′ (in exon 20)		
Phex2R	5′-TACCAGAGTCGGCAGGAATC-3′ (in exon 22)	228	
Fgf23F	5′-ACTTGTCGCAGAAGCATC-3′		
Fgf23R	5′-GTGGGCGAACAGTGTAGAA-3′	143	
Ep2F	5′-TCTCGCAGGAGAGGAGAGAG-3′		
Ep2R	5′-GGTGGCCTAAGTATGGCAAA-3′	224	
Tlr4F	5′-CAGTGGGTCAAGGAACAGAAGC-3′		
Tlr4R	5′-GACAATGAAGATGATGCCAGAGC-3′	540	[38]

### Sequencing *Phex* cDNA from ears of *Hyp-Duk/Y* mice

Four *Hyp-Duk/Y* mutants and 4*+/Y* littermates were randomly chosen. Mice were euthanised under anaesthesia (avertin 5 mg/10 g), and ears were quickly removed. Total RNA (DNA-free) was prepared using Pure-Link^TM^ Micro-to-Midi Total RNA Purification System (Invitrogen, Carlsbad, CA). cDNA was synthesised using SuperScript^TM^ First-Strand Synthesis System (Invitrogen). The *Phex* mutation in *Hyp-Duk/Y* mice is predicted to affect 2 transcripts which overlapped from exon 1 to exon15 (transcript 002 contains 15 exons and transcript 201 contains 22 exons). (http://useast.ensembl.org/Mus_musculus/Transcript/Summary?g=ENSMUSG00000057457). RT-PCR for *Phex* used primer pairs spanning the deletion sites for both transcripts: PhexF (ATGGATGCAGGGACAAAAAG, in exon 12) and PhexR01 (AAAGGCATTGACTGTTGTTGG, in exon 15) or PhexR02 (TTCCCCAAAAGAAAGGCTTC, in exon 16). Amplification conditions were 94°C, 2 min; followed by 28 cycles of 94°C for 30 s, 60°C for 40 s, and 72°C for 50 s; extension for 5 min, 72°C. Ten µl of PCR products were visualised in 2% agarose. *Gapdh* was amplified as positive control [7]. PCR products were sequenced on ABI Applied Biosystems 3730 DNA Analyzer (Life Technologies Corp., Carlsbad, CA).

**Figure 1:**
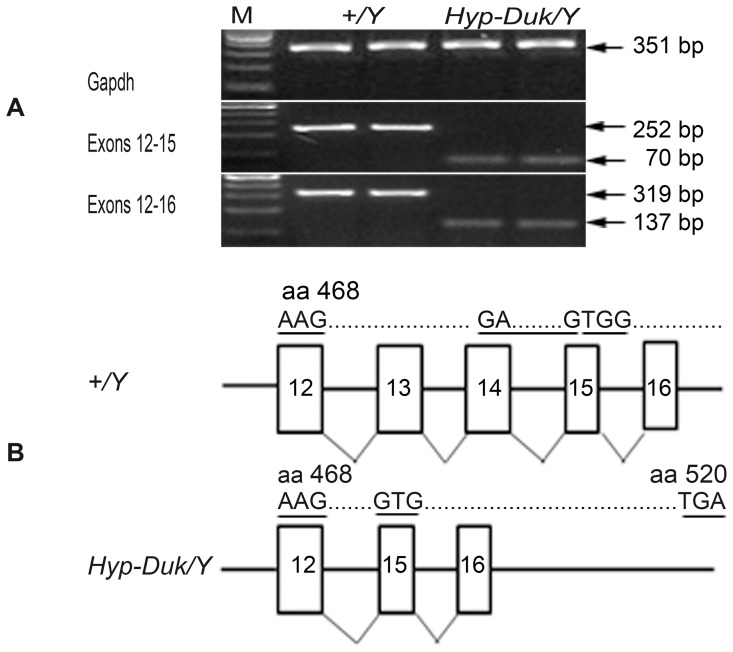
Open reading frame (ORF) shift in Phex gene of *Hyp-Duk/Y* mice. (**A**) 2% agarose gel electrophoresis of RT-PCR products from ear tissue of mutants and controls. *Gapdh* (upper panel) was amplified as a control for cDNA quality to allow comparison of expression level in the mutant and control mice. Middle (labeled exons 12–15) and lower (labeled exons 12–16) panels show the RT-PCR products using primers spanning exons 13 and 14 in two control (*+/Y*, lanes 1 and 2) and two mutant (*Hyp-Duk/Y*, lanes 3 and 4) samples. RT-PCR products from control mice were 252 bp and 319 bp, respectively, in the middle and lower panels; whereas, those from the mutant were 70 bp and 137 bp. Only a single band from each sample was amplified. M indicates 100 bp DNA ladders. (**B**) Schematic diagramme of the sequence of exons 12–16 from the wild-type mouse *Phex* gene (*+/Y*, upper panel) and the *Hyp-Duk/Y* mutant gene (lower panel). Exons 13 and 14 were missing in the *Hyp-Duk/* mouse compared with those of the control, leading to the ORF shift that occurs after aa 468 (underlined) in exon 15 and the resultant downstream stop codon at aa 520 (underlined).

### Haematoxylin/Eosin staining

Fifty-five mutant and 43 control mice from 2 to 20 weeks of age were anaesthetised and perfused through the heart left ventricle with PBS followed by Bouin's (haematoxylin/eosin or H&E staining) or by 4% paraformaldehyde (for all others) fixative. Bullae were dissected, perfused with fixative, immersed in same for 48 h, decalcified with Cal-EX solution for 6 h (or 10% EDTA, 4–7 days), embedded in paraffin, serially sectioned at 7 µm thickness, and stained with H&E for light microscopy (Leica DM4500 B, Leica Microsystems, Wetzlar, Germany) at 5 to 63× final magnification.

**Figure 2:**
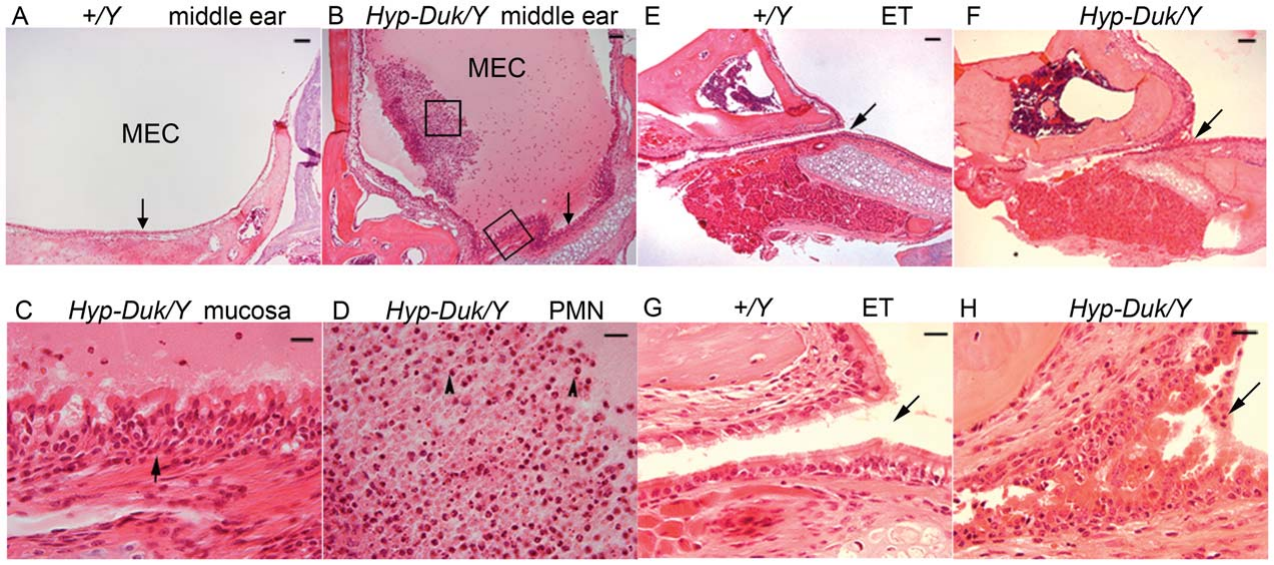
Typical middle ear pathology on H&E sections from *Hyp-Duk/Y* mice at 12 **weeks of age.** (**A**) Middle ear section shows the clear middle ear cavity (MEC) and normal epithelial layers (indicated by an arrow) in control mice (*+/Y*). (**B**) There was a large amount of effusion in the MEC and the middle ear mucosae (indicated by an arrow) were thickened in the *Hyp-Duk/Y* mice. (**C**) Enlargement from (**B**) (lower box) shows hyperplastic ciliated epithelial cells and expanded lamina propria connective tissue in the mucoperiosteum (arrow). (**D**) Enlarged from (**B**) (central box) shows the effusion material containing mainly polymorphic nuclear cells (PMN, indicated by arrowheads). Eustachian tubes (ET) as indicated by arrows appear normal in the *+/Y* mice with a single layer of epithelial cells (**E** and **G**; **G** enlarged from **E**), but exhibit narrowed and hyperplastic ciliated epithelial cells in the *Hyp-Duk/Y* mouse (**F** and **H**; **H** enlarged from **F**). Scale bars in **A, B, E**, and **F**: 100 *µm*; scale bars in C, D, G, and H: 20 *µm*.

**Table 2:** Incidence rates of otitis media in *Hyp-Duk/Y* mice by histological observation.

Age	No. of mice	No. of OM cases	Rates of OM
2 weeks	7	0	0%
3 weeks	9	4	44%
5 weeks	11	9	82%
8 weeks	8	6	75%
12 weeks	8	6	75%
≥16 weeks	12	10	83%

### Scanning electronic microscopy (SEM)

Bullae were isolated from skulls of 4 control (*+/Y*) and 4 mutant (*Hyp-Duk/Y*) mice at P21 or P150. Samples were placed in 2.5% glutaraldehyde in cacodylic acid in 0.1 M phosphate buffer (pH = 7.2) for 36 hours. After decalcification in 10% EDTA for 4–7 days, middle ear cavities were dissected from bullae and dehydrated in serial solutions of ethanol (60%, 70%, 80%, 95%, 100%) for 15 minutes each, with a second soak in 100% ethanol. Each middle ear cavity was subjected to CO_2_ critical point drying, sputter-coated with 60/40 gold-palladium, and viewed under high resolution scanning electron microscopy (Hitachi S-4500, Japan).

**Figure 3:**
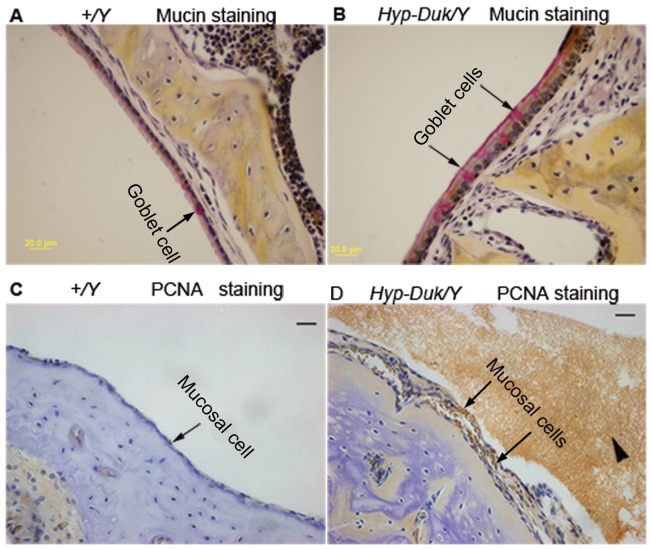
Mucin and PCNA staining reveal goblet cell and epithelial cell proliferation in middle ears of *Hyp-Duk/Y* mice. (**A, B**) Representative sections show the presence of goblet cells (indicated by arrows) in the epithelia of the middle ears of control (**A**, *+/Y*) and mutant (**B**, *Hyp-Duk/Y*) mice at 12 weeks of age. Goblet cells were found sparsely scattered among other cells in the epithelium surrounding the middle ear cavity in control mice (**A**). In contrast, goblet cells were present in relatively higher density in the middle ear epithelia of mutant mice (**B**). (**C, D**) Representative images of immunohistochemical staining of PCNA in the middle ears of control (**C**, *+/Y*) and mutant (**D**, *Hyp-Duk/Y*) mice. Strong and extensive PCNA staining appears brown in cell nuclei in the mucosal layer of the middle ear of the *Hyp-Duk/Y* mice, as indicated by arrows (**D**), implying active mucosal cell proliferation, while the mucosal cells in *+/Y* mice were comparatively negative (**C**). Middle ear effusion (arrowhead) and thickening of the epithelial layer were readily apparent (**D**). Scale bars: 20 *µm*.

### Mayer's mucicarmine method

Goblet cells in the epithelium of the middle ear mucosae were identified by Mayer's mucicarmine staining, following the protocol of Electron Microscopy Sciences (Hatfield, PA). Briefly, sections from both ears of mice at age 12 weeks (5 mutants and 4 controls, prepared as in the previous paragraph) were deparaffinised in xylene and hydrated with distilled water. Sections were first stained in Weigert's haematoxylin, approximately 7 min and washed in running water for 10 min. Second, sections were stained in mucicarmine for 60 min at room temperature and rinsed quickly in distilled water. Thirdly, sections were counterstained in metanil yellow, rinsed in distilled water for 2–4 sec and dehydrated in 95% and 100% ethanol, sequentially. After clearing in xylene, sections were mounted with coverslips using permount for microscopic observation.

**Figure 4:**
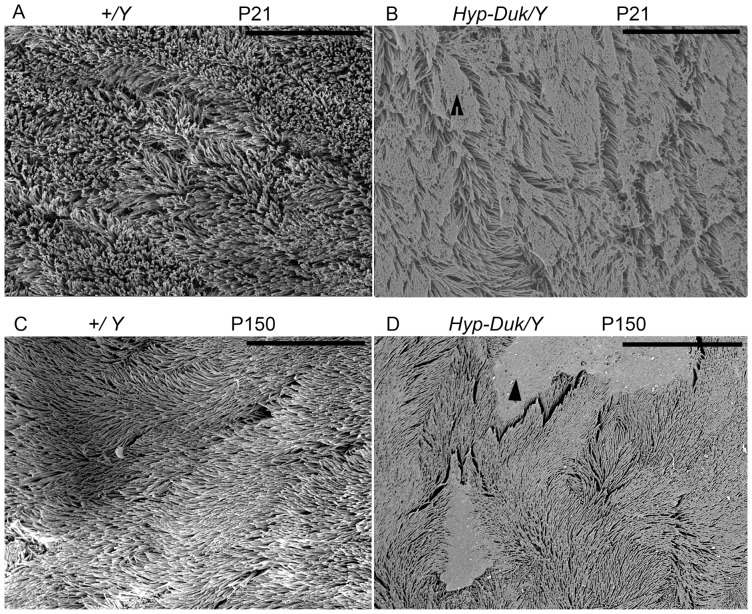
SEM observation of the cilia in the mucociliary epithelia of the middle ears from mice at P21 and P150. Wild-type littermate control (*+/Y*) mice displayed a thick lawn of morphologically normal, evenly distributed cilia in the mucociliary epithelia of the middle ears at both time points (**A, C**). The cilia of the middle ear epithelia in the *Hyp-Duk/Y* mice (**B, D**) were morphologically thinner than that of the *+/Y* mice at P21 and P150 and were irregularly distributed as a consequence of “mucin-like” material (**B**) or patches of “effusion-like” material on the top of the cilia, as indicated by arrow heads. The results indicate that *Hyp-Duk/Y* mice have difficulty clearing the effusion from the middle ears. Images are representative of each group (n = 4) at each time point. Scale bars: 15 *µm*.

### Proliferating cell nuclear antigen (PCNA) immunostaining

Specimens were prepared as described above (Mayer's Mucicarmine Method). Immunohistochemistry was performed according to standard protocols. From 4 mutant and 4 control mice at age 12 weeks, sections from both ears were deparaffinised for 15 minutes in xylene, put through two changes of 100% ethanol, and then immersed in 0.3% H_2_O_2_ in methanol for 30 min. After being washed in PBS, sections were placed in 0.2% Triton-X/PBS for 30 min. Sections were washed in PBS and blocked in 5% milk powder in PBS for 5 min. Primary antibodies (P8825, Sigma Biotechnology, 1∶3000 dilution) were applied to the slides at 4°C overnight. Anti-goat secondary antibody was applied (Sigma Biotechnology, 1∶500 dilution, incubated at room temperature for 1 h) and slides were DAB-stained (3, 3′-diaminobenzidine, Sigma) until brown cells were apparent. Reaction was quenched in PBS and sections were counterstained with haematoxylin. Slides were photographed using light microscopy (Leica, Wetzlar, Germany).

**Figure 5:**
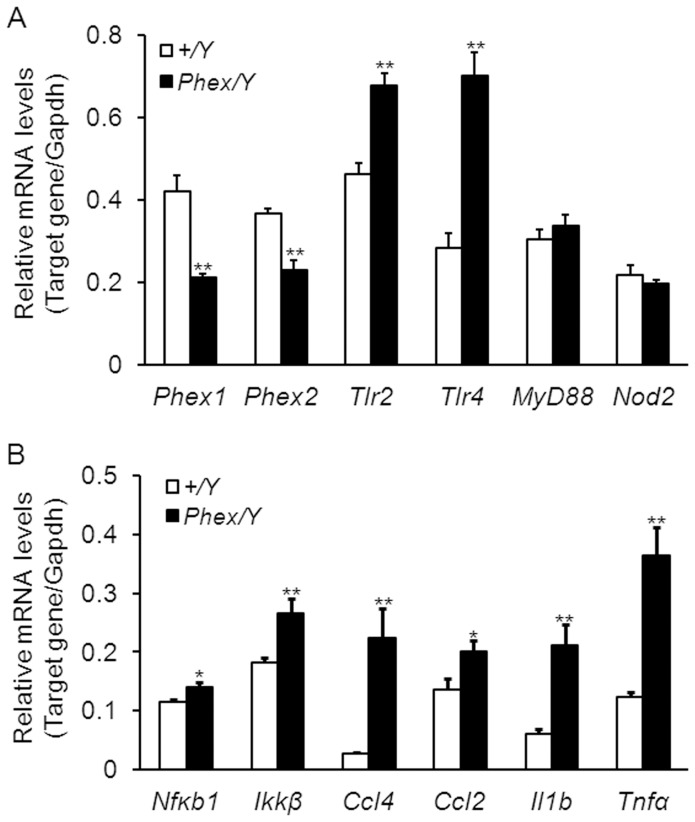
Upregulated mRNA levels of pro-inflammatory cytokines in the ears of *Hyp-Duk/Y* mice. mRNA accumulation levels from genes, mainly in the TLR-mediated pathways, were measured by Semi-quantitative RT-PCR in the ear of *Hyp-Duk/Y* mutant (n = 4) and control (*+/Y*, n = 4) mice at P35. In contrast to the downregulated mRNA levels of *Phex* gene, transcription levels of genes in TLR2- and TLR4-mediated pathways (*Nfkb1, Ikkβ Ccl4, Ccl2, Il1b* and *Tnfα*) in the ears of *Hyp-Duk/Y* mice (dark bars) were significantly higher than levels in +*/Y* mice (light bars). The mRNA levels of *MyD88* and *Nod2* were not significantly altered in the mutant mice. Error bars represent the s.e. from the mean for each sample. (** *P*<0.01; * *P*<0.05).

### Bacterial identification and antibiotic prophylaxis

Four 5-week-old mice from each genotype (*Hyp-Duk/Y* and *+/Y*) were randomly chosen and anaesthetised. Mice were euthanised by CO_2_ asphyxiation. Under sterile conditions, bullae were removed; middle ears were isolated and washed with sterile PBS. One hundred μl of each PBS lavage were inoculated onto individual BBLTM TrypticaseTM Soy Agar plates with 5% sheep blood (TSA II, Fisher, Pittsburgh, PA) and plates were incubated at 37°C, 5% CO_2_ for 18 h. Colonies were sorted, counted, and further identified. *Staphylococci* were identified by standard microbiologic features, with coagulase-negative *Staphylococci* (CNS) differentiated from *S. aureus* by positive tube-coagulase test.

**Figure 6:**
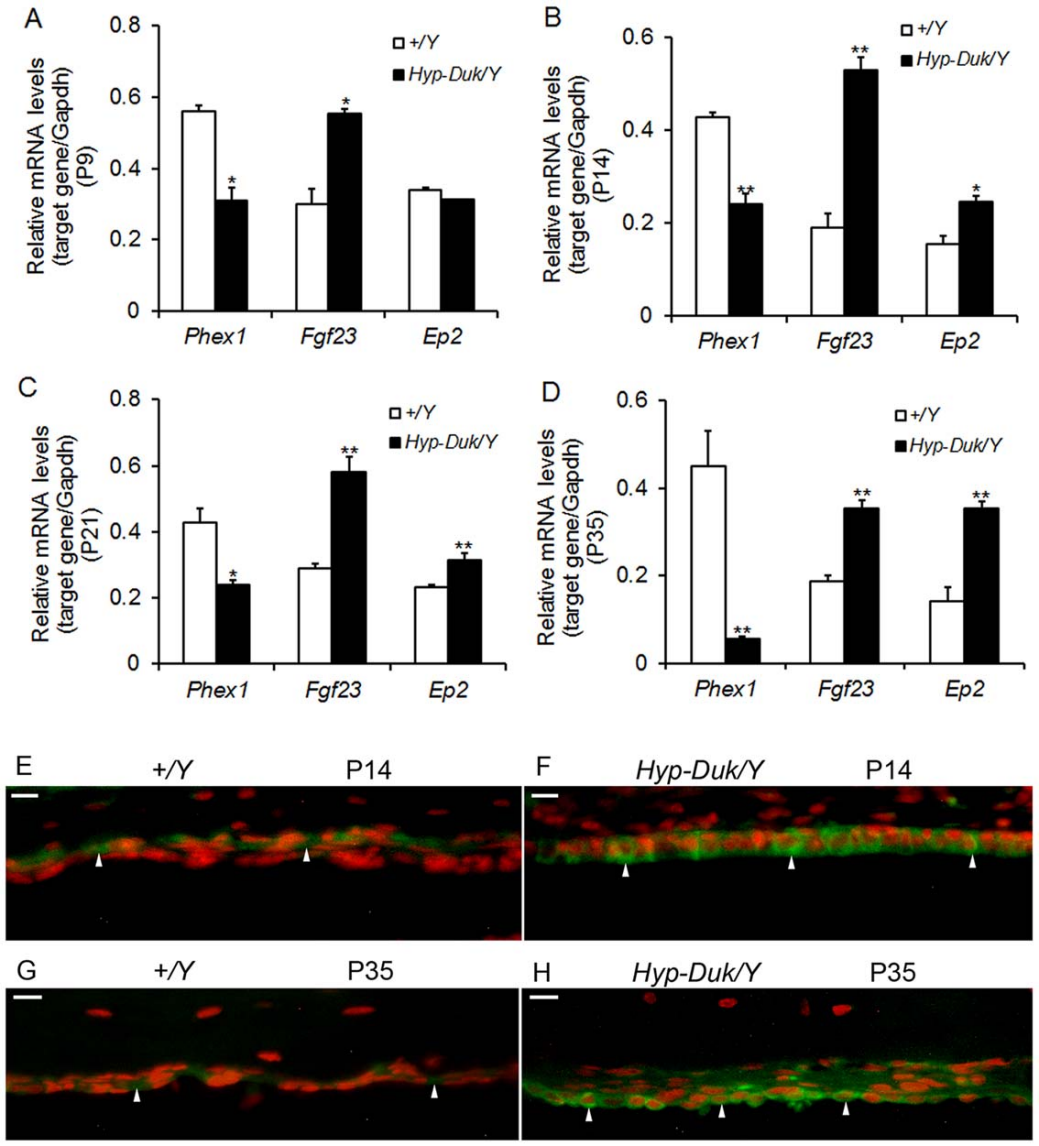
*Phex*-related gene expression in ears of *Hyp-Duk/Y* mice. (**A–D**) A time course observation of the mRNA levels of *Phex, Fgf23* and *Ep2* in the ears of *+/Y* and *Hyp-Duk/Y* mice by Semi-quantitative RT-PCR. With downregulation of *Phex* mRNA levels in the *Hyp-Duk/Y* mice, the *Fgf23* mRNA levels were corresponding increased at P9, P14, P21 and P35, whereas the mRNA levels of *Ep2* were upregulated at P14, P21 and P35, compared with the levels of *+/Y* mice. n = 3–5 for each group at each time point. Error bars represent the s.e. from the mean (*P<0.05; **P<0.01). (**E–G**) Localization of EP2 in the middle ear epithelial cells of *+/Y* and *Hyp-Duk/Y* mice at P14 and P35 by immunohistochemistry detection. Representative paraffin sections from 4*+/Y* and 4 *Hyp-Duk/Y* mice were used. The results showed that EP2 was expressed in the membrane and cytoplasm of the middle ear epithelial cells (as indicated by arrows) of both controls (**E, G**) and mutants (**F, H**) at P14 and P35, but with strong staining intensity in the *Hyp-Duk/Y* mice at both time points. Scale bar  = 10 *µm*.

Azithromycin (50 mg/kg/d) was therefore added to drinking water (final concentration of 0.25 g/l) [8] for *Phex^-Hyp-Duk^/+* mice (n = 6, mated to *+/Y* mice) at gestational day 18 (G18) and drug treatment was maintained through lactation, after which the drug was given to the litters in drinking water. At P35, antibiotic-treated control (*+/Y*, n = 4) and mutant (*Hyp-Duk*/Y, n = 5) litters were sacrificed and the bullae of ears were isolated for H&E staining and microscopy, as described in histological preparation.

**Figure 7:**
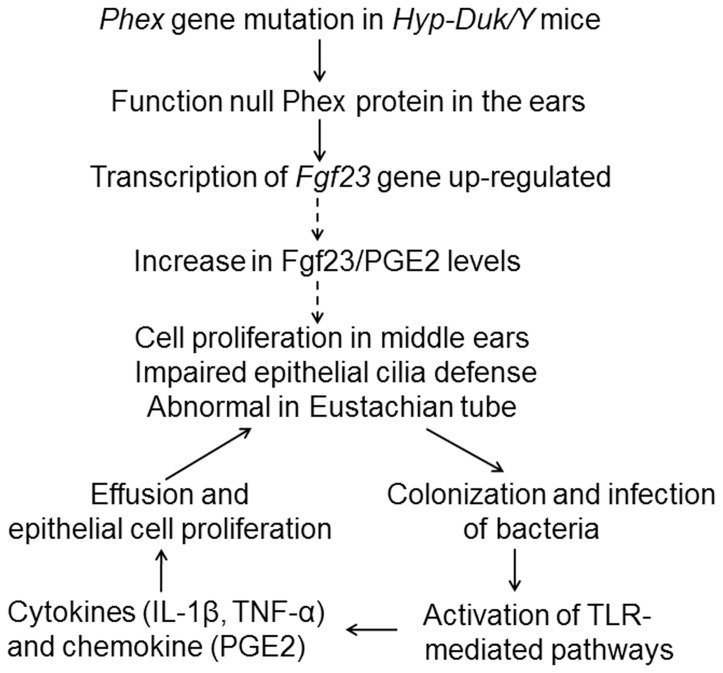
Schematic diagramme showing the hypothesised mechanism of OM development in the *Hyp-Duk/Y* mice. Possible elements or pathways involved in the OM development are illustrated. Solid lines show pathways for which there is direct evidence. Dashed lines are proposed links.

### Semi-quantitative RT-PCR for evaluation of gene transcription levels in the ears

Four to five *Hyp-Duk/Y* mutant and 3–4*+/Y* wild-type male littermates ranging in age from P9 to P35 were used for the experiments. Mouse treatment and total RNA preparation from the middle and inner ears were performed following the methods described previously and the mRNA levels of specific genes were evaluated by semi-quantitative RT-PCR [7]. Additional RT-PCR primers were listed in Table 1.

### Fluorescence immunohistochemistry (IHC) assays in middle ears

For detection of PHEX, TLR2, TLR4, NF-κB and TNF-α protein expression in the middle ears of *Hyp-Duk/Y* mice, cryosections were used and the detail methods were described in the supplementary section. To localize EP2 expression in the middle ear, paraffin sections were used following the procedure described [7]. Goat anti-EP2 polyclonal antibody (sc-22195, 200 mg/ml, Aanta Cruz Biotechnology, INC.) was used at 1∶200 dilution. Donkey anti-goat secondary antibody conjugated to Alexa Fluor-568 was used at 1∶400.

### Statistical method

*ANOVA* test was used for statistical analysis. *P*<0.05 was considered to be significant.

## Results

### Exon deletion yields functionally null PHEX protein in *Hyp-Duk/Y* mouse ears

The RT-PCR products from 2 samples in each group were randomly chosen for agarose gel electrophoresis (**Figure 1** **A**), in which one single band from each sample was amplified. Sizes of *Phex*-specific RT-PCR products from *Hyp-Duk/Y* samples were smaller (70 bp and 137 bp) than products from control mice (252 bp and 319 bp) for both primer pairs. Sequencing of RT-PCR products confirmed that exons 13 and 14 were deleted from the *Phex* cDNA, leading to an open reading frame shift after aa 468 and introducing a stop codon at aa 520 (**Figure 1** **B**). The deletion in the *Phex* gene does not alter splicing of mRNA from the mutant gene. This is the first time the *Hyp-Duk/Y* mutation has been sequenced at cDNA level to determine the specific nature of the mutation in the ears.

PHEX protein expression in the middle ears of *Hyp-Duk/Y* and control mice (P35) was determined by immunostaining with two antibodies (PHEX-H-176 and PHEX-C-13) that recognise peptides flanking the altered sequence ( aa 468 to 520) in the PHEX protein from aa positions 151 to 326 (H-176) and 530 to 560 (C-13), respectively. The antibody H-176 gave strong signals, mainly in middle ear mucosal cells, in both mutant and control mice. However, C-13 only gave signals in middle ears (mainly epithelial cells in mucosae and osteocytes in the bony wall) of control mice, and failed to stain any proteins in corresponding structures in mutant mice, indicating absence of the C-terminal portion of the PHEX protein, resulting from truncation in the mutant (Figure S1).

### Histopathologic evidence of inflammation in the middle ears of *Hyp-Duk/Y* mice

Ear sections from 55 *Hyp-Duk/Y* mice and 43 age-matched littermate control (*+/Y*) mice from 2 to 20 weeks of age were examined for histopathology. Overall, OM with effusion in the middle ear space and/ or thickened mucosae occurred in at least one ear of *Hyp-Duk/Y* mice at (or after) 3 weeks of age with an average incidence rate of 73% (35/48, Table 2). However, only two of 43 control mice at ages 3 weeks and 20 weeks, respectively, presented with OM by histological examination. In OM mice, mucosae of the middle ears and Eustachian tubes showed general thickening with proliferation of epithelial cells and lamina propria connective tissue (**Figure** **2**). The effusion was serous and contained variable numbers of inflammatory cells, mainly consisting of polymorphonuclear cells.

### Increase in goblet cells and mucosal cell proliferation in the middle ears of *Hyp-Duk/Y* mice

Sections of each ear from mutant (*Hyp-Duk/Y*) mice and control (*+/Y*) mice at age 12 weeks were stained by the Mayer's mucicarmine method to visualise goblet cells (**Figure** **3** **A, 3B**), which have been demonstrated at high density in secretory OM [9]. In control mice, goblet cells were rare, but in *Hyp-Duk/Y* mutants, goblet cells were found scattered among other cells in the middle ear cavity epithelium, especially in cuboidal ciliated epithelium. PCNA is a reliable marker of cells undergoing active proliferation [10]. Strong PCNA staining was detected in the middle ear mucosae of *Hyp-Duk/Y* mutant mice, shown in **Figure** **3D**, while epithelial cells of the middle ears in control mice were virtually PCNA negative (**Figure** **3** **C**).

### Irregular cilia on epithelial cells in middle ears of *Hyp-Duk/Y* mice

SEM images of cilia on epithelial cells in the middle ears at P21 and P150 are shown (**Figure** **4**). The control (*+/Y*) mice displayed morphologically normal, evenly distributed cilia in the mucociliary epithelia of the middle ears (**A, C**). The cilia of middle ear epithelia in the *Hyp-Duk/Y* mice (**B, D**) were morphologically thinner than those of the *+/Y* mice and were irregularly distributed at both time points. Mutation in the *Phex* gene impairs ciliary development and clearance function.

### Upregulation of pro-inflammatory cytokine expression in the middle ears of *Hyp-Duk/Y* mice

Inflammation caused by bacterial infection is mediated by TLR signaling pathways and is characterised by high level production of pro-inflammatory cytokines which contribute to otitis media pathogenesis [11]. Therefore, transcription levels of *Tlr2, Tlr4* and genes (*Ccl4, Ccl2, Il1b* and *Tnfα*) encoding pro-inflammatory cytokines were examined by semi-quantitative RT-PCR in mutant (*Hyp-Duk/Y*) and control (*+/Y*) mice. At P35, mRNA levels of the above genes were significantly upregulated (*P*<0.05 or 0.01) in the ears of mutant mice compared to those of controls (**Figure** **5**). Expression of TLR2, TLR4, NF-κB and TNF-α was also examined by in situ fluorescent IHC staining of sections from middle ears of *+/Y* and *Hyp-Duk/Y* mice at age P35 (**Figure S2**). The four proteins were mainly expressed in the middle ear mucosal layers of both mutant and control mice, whereas the staining intensity of antibodies for each of the four proteins was much stronger in middle ear mucosae of *Hyp-Duk/Y* mice than that in the middle ears of *+/Y* mice. The results indicate that TLR-mediated signaling pathways were activated in the middle ear immune response in OM.

### Pathogenic bacteria isolation from middle ears of the *Hyp-Duk/Y* mice

To investigate the causative agent of OM infection in *Hyp-Duk/Y* mice, we cultured middle ear lavage samples from 5-week-old mutant and control mice. Under microaerophilic conditions, the only type of bacteria isolated from middle ears of mutant (*Hyp-Duk/Y* ) mice were coagulase-negative *Staphylococci* (CNS), a Gram positive bacterium which has been frequently isolated from middle ear effusions in human otitis media patients [12], [13]. Though CNS were also isolated from wild-type (*+/Y*) littermates, the mean CFUs (colony-forming units) of CNS in middle ears of mutant mice (55±29.76) were significantly higher than in controls (15±15.75) (*P*<0.01). However, OM in these mice could not be prevented by azithromycin administered from G18 to P35, which was demonstrated by histological observation (data not shown).

### Upregulation of PHEX mediated gene expression in the ear of *Hyp-Duk/Y* mice

*Phex* gene mutation in *Hyp-Duk/Y* mice was previously correlated with elevated levels of fibroblast growth factor 23 (FGF23), which subsequently increases mouse prostaglandin E2 (PGE2) production in kidney [14]. Therefore, a time course determination of the transcription levels of *Phex*, *Fgf23* and *Ep2* (PGE2 receptor gene) were carried out in the mouse ears. The mRNA levels of *Phex* were significantly downregulated at P9, P14, P21 and P35 in the mutant mice, whereas those of the *Fgf23* gene were upregulated, compared with the levels of the control mice. Moreover, mRNA levels for *Ep2* were not increased in *Hyp-Duk/Y* mice at P9 and became significantly higher than controls at P14, P21 and P35 (**Figure** **6A–D**). Further fluorescent IHC assays of EP2 showed that EP2 expression was localized in the membrane and cytoplasm of the middle ear epithelial cells of both *+/Y* and *Hyp-Duk/Y* mice, but with strong staining intensity in the mutants at both P14 and P35 (**Figure** **6E–H**). The results indicate that PGE2 levels are increased in the ears of *Phex* mutants even at P14.

## Discussion

In this study, we found that *Phex* mutation predisposes the *Hyp-Duk/Y* mice to OM. The single-gene mouse model of OM is similar to Jeff (*Jf*) mice, which present with fluid and pus in the middle ear cavity [3]. Onset of OM in *Hyp-Duk/Y* mice occurs after 3 weeks of age in about 73% of *Hyp-Duk/Y* mutants (from 3 weeks to 20 weeks of age). The OM in *Hyp-Duk/Y* mice was characterised by effusion and/or mucosal cell proliferation indicated by increased goblet cell density and by PCNA staining, a marker of active cell proliferation that is associated with otitis media [15], [16]. Effusion in the middle ears attributes to local infection, activation of NF-κB and upregulated levels of cytokines [17]–[19]. Effusion can also be exacerbated by epithelial cilia deformity or by Eustachian tube constriction or obstruction [8].

Our further study implicates PHEX-FGF23 pathways in OM pathology of *Hyp-Duk/Y* mice. PHEX has been identified as causative agent of XLH and autosomal dominant hypophosphatemic rickets/osteomalacia (ADHR) [5], [20]. The native PHEX protein was reported to inactivate a phosphaturic factor, which is considered to be fibroblast growth factor 23 (FGF23) [21]. Circulatory FGF23 is elevated in most patients with XLH [22], and high level of intact FGF23 despite hypophosphatemia is a clinical indicator in XLH diagnosis [23]. While PHEX is expressed primarily in cells of bone lineage, the main protein effects on renal phosphate wasting and impaired vitamin D metabolism occur in the kidney. FGF23 regulates urinary phosphate excretion to maintain systemic phosphate homeostasis. The exact mode of action of the phosphaturic effects of FGF23 is not fully understood and is an intense area of research [24]. PHEX and the sibling protein DMP1(dentin matrix protein 1) regulate growth factor FGF23 expression in osteocytes by controlling the pathway involving FGF receptor (FGFR) signaling [25]. In cultured kidney cells, FGF23 inhibits phosphate transport [26], probably via an FGF receptor 3c which is linked to the MAPK pathway [27]. FGF23 and alpha-klotho, a transmembrane protein, together stimulated osteoblast proliferation [28]; they also increased phosphorylation of ERK1/2, P38, JNK, AKT, IκB and GSK-3β and BrdU incorporation in renal and intestinal epithelial cell lines from human [29]. Activation of FGF receptor and Wnt/β-catenin is a newly identified molecular pathway stimulated by inactivation of PHEX in osteoblasts from *Hyp* mice [30]. The above studies suggest that FGF23, by interacting with FGF receptors, can initiate downstream signaling events by multiple pathways.

FGF23 increases urinary and renal production of PGE2, which inhibits proximal tubule phosphate transport in *Hyp* mice [14]. PGE2 in different tissues may have different functions. We therefore tested whether increased FGF23 can induce production of PGE2 in the ears of *Hyp-Duk/Y* mice. Direct determination of PGE2 expression in mouse ears is not feasible; thus, transcription of PGE2 receptor (EP2) was measured. mRNA levels of *Ep2* were significantly upregulated in the ears of *Hyp-Duk/Y* mice not only at P21 and P35, but also at P14 (before the OM occurs), indicating that PGE2 would likely show a correspondent increase in the time course with OM development. As upregulation of *Fgf23* mRNA levels in the ears of *Hyp-Duk/Y* mice was correlated with the levels of EP2 from P14 to P35, we postulate that OM in *Hyp-Duk*/*Y* mice is primarily mediated by FGF23/PGE2 pathways.

PGE2 can be released from various cell types (such as macrophages and endothelial cells) and mediates inflammation through its potent vascular permeabilization activity [31]. High concentration of PGE2 in middle ear effusions has been documented and correlated with the degree of inflammation during OM in humans [32]. PGE2 increases transepithelial ion transport rate by increasing intracellular cAMP content in a middle ear cell line (MESV); such a mechanism could diminish the periciliary sol layer to impair mucociliary clearance in OM [33]. In addition, PGE2 can cause production of TNF and interleukin IL-1β, which direct neutrophil trafficking into the middle ear cavity [34]. These cytokines can exacerbate OM in *Hyp-Duk/Y* mice, as in humans, by inflammation and tissue destruction [35].

Bacterial infection is a secondary factor which augments middle ear pathology. Coagulase-negative *Staphylococci* (CNS) is currently a major cause of nosocomial and health-care related infections [36]. This bacterium was isolated from the middle ears of an OM mouse model of Down syndrome in our previous study [37]. The higher CNS density in the middle ears of *Hyp-Duk/Y* mice may trigger, at least partially, elevation of mRNA levels of pro-inflammatory cytokines in TLR2-mediated pathway. The increased TLR4 levels in the ears of *Hyp-Duk/Y* mice were probably caused by infection of Gram-negative bacteria that were unable to grow at the culture conditions. As application of azithromycin from G18 to P35 failed to prevent OM in *Hyp-Duk/Y* mice, we concluded that bacterial infection was secondary to the primary pathological alteration mediated by FGF23/PGE2 in middle ears.

In summary, *Phex* gene mutation induced primary structural and functional middle ear abnormalities and, with secondary bacterial infection, resulted in OM in the *Hyp-Duk/Y* mice (**Figure** **7**). Chronic inflammation could damage the fragile structures of the middle ear thus causing conductive and even sensory hearing loss. The *Hyp-Duk/Y* mouse is therefore a promising model for studying the molecular mechanism of OM and for testing and targeting drugs to prevent OM.

## Supporting Information

Figure S1 Deletion of exons 13–14 leading to a functionally null PHEX protein in the ears of *Hyp-Duk/Y* mice. Representative middle ear sections from control and *Hyp-Duk/Y* mice stained with anti-PHEX-H-176 and anti-PHEX-C-13 antibodies detect regions of the PHEX protein preceding and following, respectively, the deletion and consequent stop codon (revealed by Alexa Fluor 488, green). The antibody H-176 (indicated as PHEX1 above panels) revealed expression of the N-terminal region of the PHEX protein in the middle ears of both the mutant and control mice, with stronger staining in the epithelial cells of the middle ear mucosae as indicated by arrows (A and B or C and D). However, C-13 (indicated as PHEX2 above panels) showed strong positive staining in mucosal epithelial cells (indicated by the arrow at the bottom of E or F) and osteocytes (indicated by arrow in the middle of E or F) in the bony structures of the middle ears of *+/Y* mice, and failed to stain the corresponding structures in *Hyp-Duk/Y* mutant mice, indicating loss of the C-terminal portion of the PHEX protein, downstream of exon 16 (G). Panels B, D, F and H are the merged images obtained by PI (Propidium iodide, red) and antibody (green) staining. Scale bars: 20 *μm*.
(TIF)

Figure S2 Representative IHC staining the sections of middle ears from *+/Y* and *Hyp-Duk/Y* mice at the age of 5 weeks. (A to D) Anti-TLR2–FITC staining of middle ears from mice of *+/Y* (A), *Hyp-Duk/Y* (B), *Hyp-Duk/Y* with primary antibody omission (C) and *TLR2^−/−^* (D). (E, F) Anti-TLR4–FITC staining of the middle ears from mice of *+/Y* (E) and *Hyp-Duk/Y* (F); (G, H) are enlarged area from (F) to show TLR4 expression in PMNs in the middle ear indicated by arrows. (I and J, K and L) Anti-NFκB–FITC and anti-TNFα–FITC staining of middle ear from *+/Y* and *Hyp-Duk/Y* mice, respectively. Overall, TLR2, TLR4, NF-κB and TNF-α were expressed in the middle ear mucosae of both control and mutant mice and the staining intensity of the 4 antibodies was much stronger in mucosae of the *Hyp-Duk/Y* mice than in the middle ears of the *+/Y* mice as indicated by the arrows. Scale bars: 100 *µm* in (A–F, I–J) and 20 *µm* in (G, H).
(TIF)

Materials and Methods S1 (DOC)

## References

[1] Rovers MM, Schilder AG, Zielhuis GA, Rosenfeld RM (2004) Otitis media. Lancet 363: 465–473. 14962529 10.1016/S0140-6736(04)15495-0

[2] Darrow DH, Dash N, Derkay CS (2003) Otitis media: concepts and controversies. Curr Opin Otolaryngol Head Neck Surg 11: 416–423. 14631172 10.1097/00020840-200312000-00002

[3] Hardisty RE, Erven A, Logan K, Morse S, Guionaud S, et al (2003) The deaf mouse mutant Jeff (Jf) is a single gene model of otitis media. J Assoc Res Otolaryngol 4: 130–138. 12943368 10.1007/s10162-002-3015-9 PMC3202714

[4] Tournis ST, Giannikou PV, Paspati IN, Katsalira EA, Voskaki IC, et al (2005) Co-existence of X-linked hypophosphatemic rickets (XLH) and primary hyperparathyroidism: case report and review of the literature. J Musculoskelet Neuronal Interact 5: 150–154. 15951631

[5] Lorenz-Depiereux B, Guido VE, Johnson KR, Zheng QY, Gagnon LH, et al (2004) New intragenic deletions in the Phex gene clarify X-linked hypophosphatemia-related abnormalities in mice. Mamm Genome 15: 151–161. 15029877 10.1007/s00335-003-2310-z PMC2859190

[6] Megerian CA, Semaan MT, Aftab S, Kisley LB, Zheng QY, et al (2008) A mouse model with postnatal endolymphatic hydrops and hearing loss. Hear Res 237: 90–105. 18289812 10.1016/j.heares.2008.01.002 PMC2858221

[7] Han F, Yu H, Tian C, Li S, Jacobs MR, et al (2009) Role for Toll-like receptor 2 in the immune response to Streptococcus pneumoniae infection in mouse otitis media. Infect Immun 77: 3100–3108. 19414550 10.1128/IAI.00204-09 PMC2708554

[8] Depreux FF, Darrow K, Conner DA, Eavey RD, Liberman MC, et al (2008) Eya4-deficient mice are a model for heritable otitis media. J Clin Invest 118: 651–658. 18219393 10.1172/JCI32899 PMC2213371

[9] Tos M, Bak-Pedersen K (1975) Density of goblet cells in chronic secretory otitis media: findings in a biopsy material. Laryngoscope 85: 377–383. 1113605 10.1288/00005537-197502000-00015

[10] Furukawa M, Ebmeyer J, Pak K, Austin DA, Melhus A, et al (2007) Jun N-terminal protein kinase enhances middle ear mucosal proliferation during bacterial otitis media. Infection and Immunity 75: 2562–2571. 17325051 10.1128/IAI.01656-06 PMC1865762

[11] Smirnova MG, Birchall JP, Pearson JP (2004) The immunoregulatory and allergy-associated cytokines in the aetiology of the otitis media with effusion. Mediators Inflamm 13: 75–88. 15203548 10.1080/09629350410001688477 PMC1781541

[12] Bunse T, Hildmann H, Zan W, Opferkuch W (1987) An immunological study of otitis media with effusion. Antibodies directed against coagulase-negative staphylococci in the effusion fluid. Arch Otorhinolaryngol 244: 123–126. 3662924 10.1007/BF00458562

[13] Bunse T, Hildmann H, Zan W, Opferkuch W (1987) A bacteriological study of otitis media with effusion. Concurrent coagulase-negative staphylococcal infections in the middle ear. Arch Otorhinolaryngol 243: 387–391. 3566622 10.1007/BF00464648

[14] Syal A, Schiavi S, Chakravarty S, Dwarakanath V, Quigley R, et al (2006) Fibroblast growth factor-23 increases mouse PGE2 production in vivo and in vitro. Am J Physiol Renal Physiol 290: F450–455. 16144964 10.1152/ajprenal.00234.2005 PMC4097041

[15] Lim DJ, Birck H (1971) Ultrastructural pathology of the middle ear mucosa in serous otitis media. Ann Otol Rhinol Laryngol 80: 838–853. 5127754 10.1177/000348947108000611

[16] Lim DJ, Klainer A (1971) Cellular reactions in acute otitis media–scanning and transmission electron microscopy. Laryngoscope 81: 1772–1786. 5117718 10.1288/00005537-197111000-00003

[17] Kubba H, Pearson JP, Birchall JP (2000) The aetiology of otitis media with effusion: a review. Clin Otolaryngol 25: 181–194. 10944048 10.1046/j.1365-2273.2000.00350.x

[18] Schachern PA, Tsuprun V, Cureoglu S, Ferrieri P, Briles DE, et al (2009) Virulence of pneumococcal proteins on the inner ear. Arch Otolaryngol Head Neck Surg 135: 657–661. 19620586 10.1001/archoto.2009.72

[19] Samuel EA, Burrows A, Kerschner JE (2008) Cytokine regulation of mucin secretion in a human middle ear epithelial model. Cytokine 41: 38–43. 18063379 10.1016/j.cyto.2007.10.009 PMC2255598

[20] Erben RG, Mayer D, Weber K, Jonsson K, Juppner H, et al (2005) Overexpression of human PHEX under the human beta-actin promoter does not fully rescue the Hyp mouse phenotype. J Bone Miner Res 20: 1149–1160. 15940367 10.1359/JBMR.050212

[21] Fukumoto S, Yamashita T (2002) Fibroblast growth factor-23 is the phosphaturic factor in tumor-induced osteomalacia and may be phosphatonin. Curr Opin Nephrol Hypertens 11: 385–389. 12105387 10.1097/00041552-200207000-00003

[22] Imanishi Y (2002) [FGF-23 on hypophosphatemic rickets/osteomalacia]. Clin Calcium 12: 634–637. 15775350

[23] Igaki JM, Yamada M, Yamazaki Y, Koto S, Izawa M, et al. High iFGF23 level despite hypophosphatemia is one of the clinical indicators to make diagnosis of XLH. Endocr J 58: 647–655. 21597229 10.1507/endocrj.k10e-257

[24] Razzaque MS (2009) FGF23-mediated regulation of systemic phosphate homeostasis: is Klotho an essential player? Am J Physiol Renal Physiol 296: F470–476. 19019915 10.1152/ajprenal.90538.2008 PMC2660189

[25] Martin A, Liu S, David V, Li H, Karydis A, et al. Bone proteins PHEX and DMP1 regulate fibroblastic growth factor Fgf23 expression in osteocytes through a common pathway involving FGF receptor (FGFR) signaling. FASEB J 25: 2551–2562. 10.1096/fj.10-177816 PMC3136343 21507898

[26] Bowe AE, Finnegan R, Jan de Beur SM, Cho J, Levine MA, et al (2001) FGF-23 inhibits renal tubular phosphate transport and is a PHEX substrate. Biochem Biophys Res Commun 284: 977–981. 11409890 10.1006/bbrc.2001.5084

[27] Yamashita T, Konishi M, Miyake A, Inui K, Itoh N (2002) Fibroblast growth factor (FGF)-23 inhibits renal phosphate reabsorption by activation of the mitogen-activated protein kinase pathway. J Biol Chem 277: 28265–28270. 12032146 10.1074/jbc.M202527200

[28] Shalhoub V, Ward SC, Sun B, Stevens J, Renshaw L, et al. Fibroblast growth factor 23 (FGF23) and alpha-klotho stimulate osteoblastic MC3T3.E1 cell proliferation and inhibit mineralization. Calcif Tissue Int 89: 140–150. 21633782 10.1007/s00223-011-9501-5 PMC3135830

[29] Medici D, Razzaque MS, Deluca S, Rector TL, Hou B, et al (2008) FGF-23-Klotho signaling stimulates proliferation and prevents vitamin D-induced apoptosis. J Cell Biol 182: 459–465. 18678710 10.1083/jcb.200803024 PMC2500132

[30] Liu S, Tang W, Fang J, Ren J, Li H, et al (2009) Novel regulators of Fgf23 expression and mineralization in Hyp bone. Mol Endocrinol 23: 1505–1518. 19556340 10.1210/me.2009-0085 PMC2737552

[31] Enomoto F, Ichikawa G, Nagaoka I, Yamashita T (1995) [Evaluation of arachidonic acid metabolites in experimental rat otitis media with effusion]. Nippon Jibiinkoka Gakkai Kaiho 98: 959–967. 7629649 10.3950/jibiinkoka.98.959

[32] Jung TT (1988) Prostaglandins, leukotrienes, and other arachidonic acid metabolites in the pathogenesis of otitis media. Laryngoscope 98: 980–993. 2842558 10.1288/00005537-198809000-00013

[33] Herman P, Yen PT, Tu TY, Loiseau A, Cassingena R, et al (1994) Pathophysiology of middle ear epithelium: a new role for prostaglandin E2. Am J Otolaryngol 15: 258–266. 7978024 10.1016/0196-0709(94)90092-2

[34] Skovbjerg S, Roos K, Nowrouzian F, Lindh M, Holm SE, et al. High cytokine levels in perforated acute otitis media exudates containing live bacteria. Clin Microbiol Infect 16: 1382–1388. 10.1111/j.1469-0691.2010.03083.x PMC7128526 19832705

[35] Dinarello CA (2000) Proinflammatory cytokines. Chest 118: 503–508. 10936147 10.1378/chest.118.2.503

[36] Piette A, Verschraegen G (2009) Role of coagulase-negative staphylococci in human disease. Vet Microbiol 134: 45–54. 18986783 10.1016/j.vetmic.2008.09.009

[37] Han F, Yu H, Zhang J, Tian C, Schmidt C, et al (2009) Otitis media in a mouse model for Down syndrome. Int J Exp Pathol 90: 480–488. 19765102 10.1111/j.1365-2613.2009.00677.x PMC2768146

[38] Tang SC, Arumugam TV, Xu X, Cheng A, Mughal MR, et al (2007) Pivotal role for neuronal Toll-like receptors in ischemic brain injury and functional deficits. Proc Natl Acad Sci U S A 104: 13798–13803. 17693552 10.1073/pnas.0702553104 PMC1959462

