# Supplementary figures and images for: Mutation in Phex Gene Predisposes BALB/c-PhexHyp-Duk/Y Mice to Otitis Media
Source: PLoS One. 2012 Sep 28;7(9):e43010. doi: 10.1371/journal.pone.0043010 (PMC3461009; doi:10.1371/journal.pone.0043010)

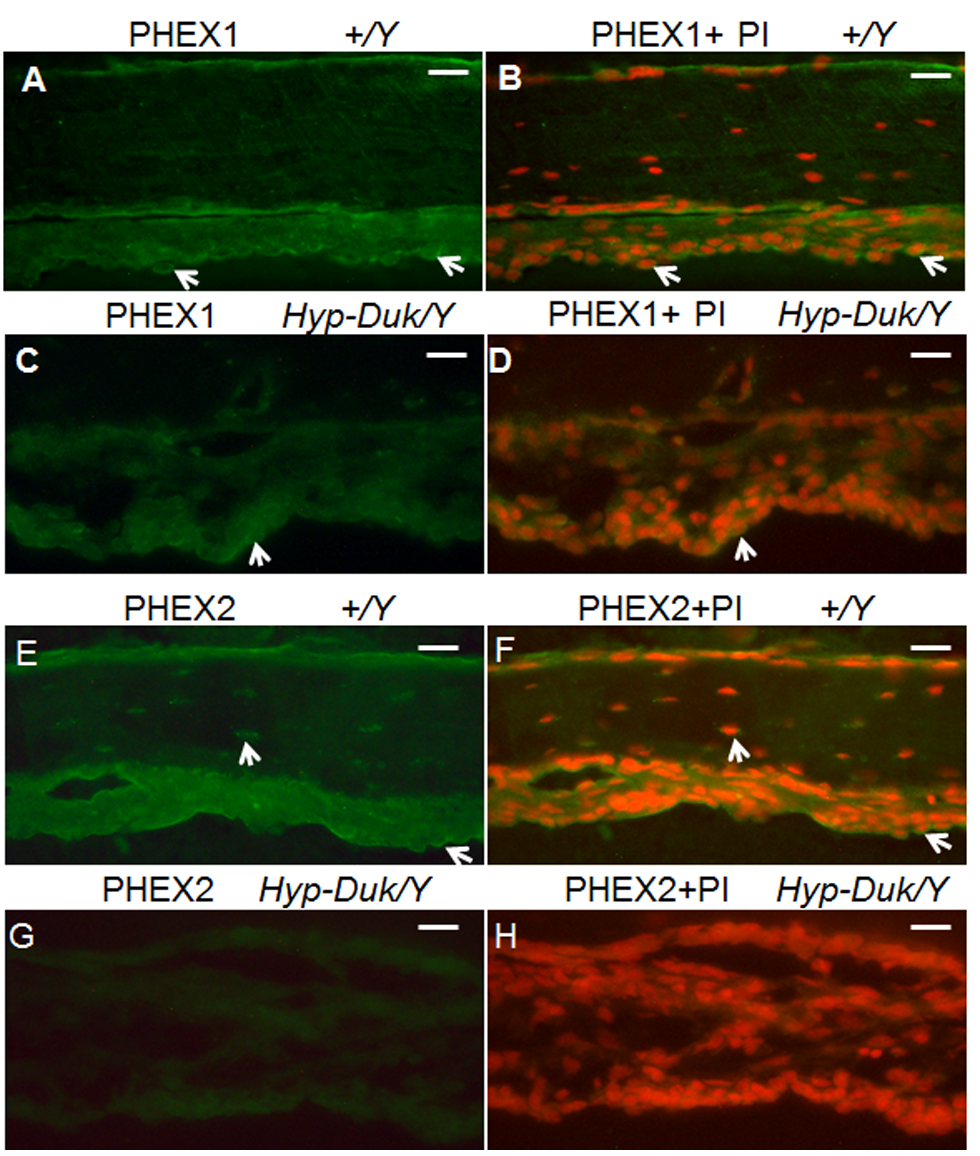

Supplement: Figure S1 — Deletion of exons 13–14 leading to a functionally null PHEX protein in the ears of Hyp-Duk/Y mice. Representative middle ear sections from control and Hyp-Duk/Y mice stained with anti-PHEX-H-176 and anti-PHEX-C-13 antibodies detect regions of the PHEX protein preceding and following, respectively, the deletion and consequent stop codon (revealed by Alexa Fluor 488, green). The antibody H-176 (indicated as PHEX1 above panels) revealed expression of the N-terminal region of the PHEX protein in the middle ears of both the mutant and control mice, with stronger staining in the epithelial cells of the middle ear mucosae as indicated by arrows (A and B or C and D). However, C-13 (indicated as PHEX2 above panels) showed strong positive staining in mucosal epithelial cells (indicated by the arrow at the bottom of E or F) and osteocytes (indicated by arrow in the middle of E or F) in the bony structures of the middle ears of +/Y mice, and failed to stain the corresponding structures in Hyp-Duk/Y mutant mice, indicating loss of the C-terminal portion of the PHEX protein, downstream of exon 16 (G). Panels B, D, F and H are the merged images obtained by PI (Propidium iodide, red) and antibody (green) staining. Scale bars: 20 μm. (TIF) [file pone.0043010.s001.tif]

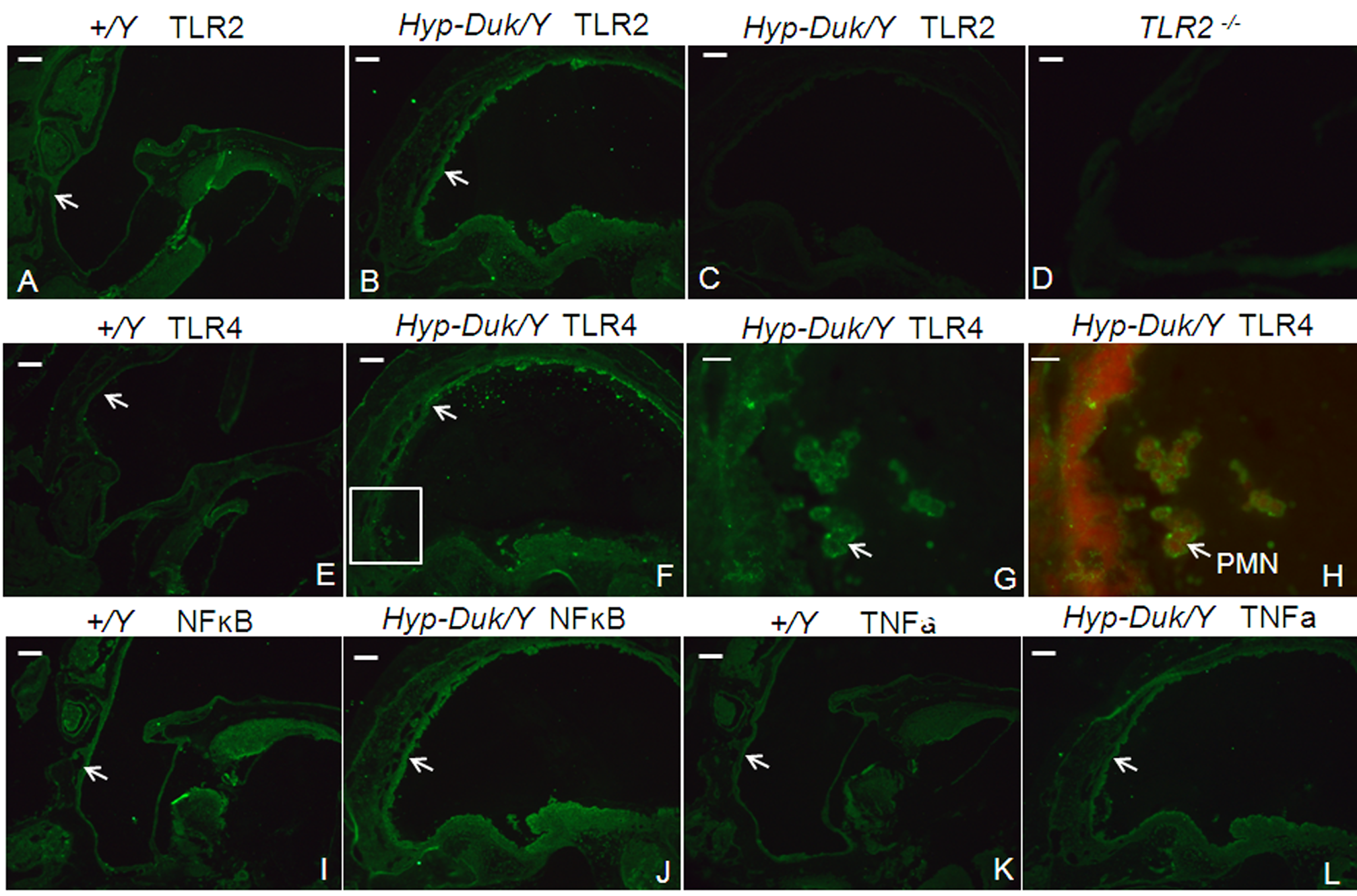

Supplement: Figure S2 — Representative IHC staining the sections of middle ears from +/Y and Hyp-Duk/Y mice at the age of 5 weeks. (A to D) Anti-TLR2–FITC staining of middle ears from mice of +/Y (A), Hyp-Duk/Y (B), Hyp-Duk/Y with primary antibody omission (C) and TLR2−/− (D). (E, F) Anti-TLR4–FITC staining of the middle ears from mice of +/Y (E) and Hyp-Duk/Y (F); (G, H) are enlarged area from (F) to show TLR4 expression in PMNs in the middle ear indicated by arrows. (I and J, K and L) Anti-NFκB–FITC and anti-TNFα–FITC staining of middle ear from +/Y and Hyp-Duk/Y mice, respectively. Overall, TLR2, TLR4, NF-κB and TNF-α were expressed in the middle ear mucosae of both control and mutant mice and the staining intensity of the 4 antibodies was much stronger in mucosae of the Hyp-Duk/Y mice than in the middle ears of the +/Y mice as indicated by the arrows. Scale bars: 100 µm in (A–F, I–J) and 20 µm in (G, H). (TIF) [file pone.0043010.s002.tif]
