# Supplementary material for: Mutation in Phex Gene Predisposes BALB/c-PhexHyp-Duk/Y Mice to Otitis Media
Source: PLoS One. 2012 Sep 28;7(9):e43010. doi: 10.1371/journal.pone.0043010 (PMC3461009; doi:10.1371/journal.pone.0043010)
Supplement: Materials and Methods S1 — (DOC) [file pone.0043010.s003.doc]

**Supplementary materials and methods**

**1. Fluorescence immunohistochemistry (IHC) assays for PHEX in mouse middle ears**

For PHEX protein expression in ears**,** 5 *Hyp-Duk/Y* mutants and 5 *+/Y* littermates at age 5 weeks were euthanised by CO_2_ asphyxiation. Bullae were dissected (through a small perforation in the inner ear apex) and fixed in 4% paraformaldehyde (in 0.1 M sodium phosphate buffer, pH 7.4, PBS) overnight, 4°C. Bullae were decalcified in 10% EDTA, 4°C, 12 h, infiltrated in gradient sucrose (10%, 20% and 30% in PBS), 4°C for 3 h, 3 h and 12 h, respectively, and embedded in OCT, 4°C, overnight. Cryosections of 7 µm thickness were immersed in freshly prepared 4% paraformaldehyde, 10 min. After two 5-min washes in 1× PBS, sections were immersed in 0.2% Triton X-100, 5 min. After another two washes in 1× PBS and blocking in 3% goat (or rabbit) serum and 2% BSA, 1 h, samples were immersed in rabbit (or goat) anti-mouse polyclonal antibody (2.00 µg/ml; 1:200 dilution), 4°C, overnight. The rabbit anti-PHEX- (H-176, sc-98996) was raised against amino acids (aa, 151 to 326) of PHEX, the goat anti-PHEX (C-13, sc-47324) was raised against peptides (aa 530-560) of PHEX; both were affinity purified (Santa Cruz Biotechnology, Inc.). Following primary antibody incubation, sections were washed twice in 1× PBS, immersed in goat anti-rabbit (or rabbit anti-goat) conjugated to Alexa Fluor 488 (1:500 dilution; Invitrogen), 1h, room temperature. Samples were mounted in VECTASHIELD® Mounting Medium (Vector Laboratories, Inc., Burlingame, CA) and observed under immunofluorescence microscopy (Leica, DM4500 B, Leica Microsystems, Wetzlar, Germany). Images were acquired at 5 to 63× magnification and analyzed using MetaMorph software (MDS Analytical Technologies).

**2. Fluorescent immunohistochemical (IHC) staining of TLR2, TLR4, NF-ĸB and TNF-α in the mouse middle ears**

Five *Hyp-Duk/Y* mutants and 5 *+/Y* littermates at age of 5 weeks were cryosectioned and IHC immunostained as in the assays of PHEX in middle ears. Rabbit anti-mouse polyclonal antibodies were purchased from Abcam, Inc. (Cambridge, MA). TLR2 antibodies (ab24192, 1.000 mg/ml) and ab47093 TLR4 antibodies (ab47093, 0.5000 mg/ml ) were used at 1:400 dilution. NF-ĸB p105/p50 antibodies (ab19285, 1.000 mg/ml) and tumour necrosis factor alpha (TNF-α) antibodies (ab9739, 0.500 mg/ml) were used at 1:200 dilution. Goat anti-rabbit secondary antibody conjugated to Alexa Fluor 488 was used for fluorescent signal development.
